# Association Between Medication-Related Problems and Emergency Room Visits Among Community-Dwelling Older Adults

**DOI:** 10.1093/geroni/igaa057.1401

**Published:** 2020-12-16

**Authors:** YuJun Zhu, Marsha Meyer, Denise Likar, Susan Enguidanos

**Affiliations:** 1 University of Southern California, Los Angeles, California, United States; 2 SCAN Health Plan, Long Beach, California, United States

## Abstract

Medication-related problems (MRPs) remain one of the largest health risks for older adults, yet there are few studies that identify the complete myriad of problems associated with medication use among community-dwelling older adults with chronic diseases. The aim of this study is to identify the range and quantity of MRPs among community-dwelling older adults and determine the relationship between number of MRPs and emergency room (ER) visits. Primary data were collected from a community medication program for diverse older adults (N=206). A comprehensive medication review was conducted to identify MRPs including adverse drug reaction, inappropriate storage, and non-adherence. We conducted multivariate logistic regression to examine the relationship between the number of MRPs and ER visits controlling for health conditions and demographic information including age, gender and race. The mean age of participants was 74.2 (SD=8.8) and 65% were female. Racial groups include Whites (39%), Blacks (21%), Hispanics (29%) and Asian/Pacific Islanders (11%). On average, participants had 5.3 (SD=2.3) health conditions and 18.8 (SD=5.9) MRPs, among which adherence (36%) and coordination of care (28%) were most commonly identified. About 30% had at least 1 ER visit in the previous 6 months. The number of MRPs was significantly associated with ER visits (OR=1.08, p=.03). Older adults experienced a variety of MRPs and for each problem experienced, the odds of having an ER visit increased by 8%. Interventions are needed to assess the myriad of MRPs among community-dwelling older adults and address those potential risks.

